# Cognitive Development Is a Reconstruction Process that May Follow Different Pathways: The Case of Number

**DOI:** 10.3390/jintelligence6010015

**Published:** 2018-03-08

**Authors:** Jacques Lautrey

**Affiliations:** Institut de Psychologie, Paris Descartes University, 71 Avenue Edouard Vaillant, 92774 Boulogne-Billancourt, France; jacques.lautrey@wanadoo.fr

**Keywords:** cognitive development, number, numerical cognition, individual differences, variability

## Abstract

Some cognitive functions shared by humans and certain animals were acquired early in the course of phylogeny and, in humans, are operational in their primitive form shortly after birth. This is the case for the quantification of discrete objects. The further phylogenetic evolution of the human brain allows such functions to be reconstructed in a much more sophisticated way during child development. Certain functional characteristics of the brain (plasticity, multiple cognitive processes involved in the same response, interactions, and substitution relationships between those processes) provide degrees of freedom that open up the possibility of different pathways of reconstruction. The within- and between-individual variability of these developmental pathways offers an original window on the dynamics of development. Here, I will illustrate this theoretical approach to cognitive development—which can be called “reconstructivist” and “pluralistic”—using children’s construction of number as an example.

## 1. Introduction

The objective of every science is to uncover the invariants that underlie the variability of observable phenomena occurring in its domain. Each science nevertheless makes epistemological choices that are specific to it. In doing so, it makes a distinction among the various forms of variability to which it is confronted, between those it deems relevant to its object of study and those it sees as bothersome. The latter, which it decides to ignore or control in one way or another, by averaging for example, are often the ones that cannot be interpreted in the framework of current theoretical paradigms [1].

The major theoretical paradigms that dominated developmental psychology in the past—Piagetian constructivism starting in the 1950s, and neo-nativism starting in the 1960s—did not lend themselves to explaining variability phenomena. In positing that deep cognitive structures are neither general nor constructed but domain-specific and innate, Chomsky’s theory differs fundamentally from Piaget’s, granted, but resembles it in its quest for universality. Both the constructivist approach and the nativist approach were aimed at uncovering the deep cognitive structures that characterize the human species and are invariant across eras, individuals, and cultures. In the end, it was the structuralist approach that led, in both cases, to the search for what is common to all individuals, and that considered between- and within-individual variabilities to be irrelevant to this object of study.

If more recent theories of cognitive development are more interested in variability, it is probably because they are inspired by theoretical paradigms in which fluctuations, within- and between-individual variabilities are not seen as bothersome background noise that can be ignored when extracting the general laws of cognitive development but, on the contrary, as an essential ingredient of evolution and change. This is the case of theories inspired by dynamic systems modeling [2,3,4], connectionist modeling [5,6], and theories that make use of the conceptual framework of Darwin’s theory of evolution, to model developmental changes [7]. This is also the case of theories that seek to integrate the contributions of the factorial approach to intelligence, based on individual differences, and the developmental approach to cognition [8,9,10].

The pluralistic approach to the relationships between cognitive development and variabilities proposed in this article is part of this trend. It proposes a conceptual framework capable of integrating both the general and the variable into cognitive development.

## 2. Conceptual Framework

The main concepts underlying this approach are reconstruction, plurality, interaction, and substitution. Each of these concepts is explicated below and will be illustrated in a concrete way using the example of the development of numerical quantification in children.

### 2.1. The Notion of Reconstruction

Cognitive development is not seen as a product of innate structures, nor as a de novo construction process, but as a process by way of which primitive functions are reconstructed. The neo-nativist trend gave rise to studies on the cognitive abilities of infants and contributed to a complete renewal of knowledge in this field. Many studies using methods suited to the capacities of infants showed that certain behaviors regarded by Piaget as indicators of the acquisition of new structures had been observed much earlier in development. It was sometimes concluded that early infant abilities evidenced in this way are underlain by the same cognitive structures as those found in older children, and hence, that these structures are innate. My own hypothesis about the resemblance between infants’ abilities and those of older children is that it does not originate in the fact that they have the same underlying cognitive structure, but in the fact that they perform the same function at both ages. The major cognitive functions that enable living beings to adapt to their environment—for example, categorizing, quantifying, orienting oneself in space, and communicating—were selected in the course of phylogenesis and were gradually integrated into the genetic heritage of certain animal species, including the human species. In this primitive form, they are operational soon after birth. But the cognitive structures in which they are rooted are different in nature from those that will be reconstructed during development. In humans, the further phylogenetic evolution of the brain endowed the species with other capacities, such as symbolic representation and cognitive control. One can assume that in situations where the primitive function is elicited, the underlying neural structure acts as an attractor around which will aggregate groups of neurons that are likely to perform the function in a more reliable and efficient way using other means.

### 2.2. The Notion of Plurality

As the reconstruction process takes place, a system is constituted that aggregates all cognitive processes capable of performing a given function. In the end, it is a plurality of processes that get activated to fulfill one and the same function, but not all of these processes will necessarily treat the same information. Some will be more suited to treating certain situations; some will be preferred by certain individuals. As we shall see below, processing plurality gives degrees of freedom to cognitive functioning and provides several possible pathways toward the reconstruction of the function [11,12,13].

### 2.3. The Notion of Interaction

If the different processes activated to fulfill a given function interact, then—insofar as they do not all process the same information—each one can transmit to each of the others, either directly or via a common interface, information that is not available to the other processes. When this occurs, the conditions are satisfied for the joint generation of a system in which the functioning of each process affects the functioning of each of the others. Models of such dynamic systems have shown that a system with these characteristics can be a source of self-organization. In the approach to development based on dynamic-system modeling [2,4,14], this kind of self-organization is seen as one of the potential sources of developmental change. Several types of interaction are possible (e.g., complementarity, competition, mutual support). The type considered here is an interaction of mutual support or reciprocal causality that is particularly conducive to initiating an improving self-organization process. For an example, see van der Maas et al. [15], who simulated the self-development of a system with five initially unrelated but mutually supportive components. There are, of course, other sources of development, such as the myelination of neural structures, but interactions between processes capable of carrying out a given function can be another source of change.

### 2.4. The Notion of Substitution^1^

Whenever several cognitive processes are capable of fulfilling the same function, a certain amount of redundancy is generated in the system they form, and this offers some functional degrees of freedom. Consequently, if one of the processes in the system is damaged, another can compensate for its absence—either partially or fully—by performing the shared function. In this case, we speak of compensatory relationships between processes. Substitution possibilities may also exist when none of the co-functional processes are damaged but have different probabilities of activation, depending on the individual and the situation. Reuchlin [16] proposed a probabilistic model of substitution relationships between cognitive processes. It postulates the existence of an activation-probability hierarchy that ranks the processes that perform the same function. The hierarchy is not necessarily the same for all individuals confronted with the same situation. This is a source of *between-individual variability* in the nature of the cognitive processes activated to accomplish the task. Symmetrically, the hierarchy may not be the same, for a given individual, in different situations or at different moments in time. It will depend on the degree of situational affordance of each process. This is a source of *within-individual variability*. Substitution relationships between processes give plasticity to the cognitive system and contribute to its reliability. They also help account for—and this is our key point of interest here—between- and within-individual variations in a general model of development [12,13].

The conceptual framework presented above is theoretical and largely hypothetical. In what follows, I will try to demonstrate its validity using the quantification of sets of discrete objects as an example to illustrate and concretize the conceptual framework proposed.

## 3. Initial State of Numerical Cognition Development

Two non-verbal, non-symbolic systems via which infants quantify sets of discrete objects have been brought to the fore. The first, called the “Approximate Number System” (ANS), provides an approximate, noisy estimate of the numerosity of large sets of objects. The second, called the “Parallel Individuation System” (PIS), gives an exact quantification of small sets of no more than three or four objects. Only the main characteristics of these two systems will be presented here. More detailed descriptions can be found elsewhere [17,18].

### 3.1. The Approximate Number System (ANS)

The function of the ANS system of numerical information processing is to provide an approximate estimate of the numerosity of a set of perceived objects. In the experiments designed to study its properties, the stimuli are sets of points or objects. The number of items in the set is varied while controlling the continuous variables likely to co-vary with that number (total area occupied, total perimeter, density). The task consists of discriminating or comparing the number of points or objects presented. With infants, the experimental paradigm used is habituation [19]. With older children or adults, the task usually consists of having the participant point to the greater of the two numerosities presented. In this case, the experimenter ensures that the display time is too short to allow the participant to count [20]. With primates, this same task is presented using conditioning methods [21].

In all cases, whether with humans (infants or adults) or apes, the participants prove capable of approximately estimating the number of items to discriminate or compare, provided the difference in number is large enough. The larger the to-be-distinguished numerosities, the noisier the estimates and hence, the farther away from each other the numerosities must be to be perceived. The ratio between this distance and the set size nevertheless remains constant. More specifically, the approximate estimation of numerosity obeys Weber’s law: the estimates are a logarithmic function of the real numerosity. This logarithmic function is considered to be the signature of the ANS, and Weber’s fraction^2^ tells us about the acuity of the ANS, i.e., the smallest difference in numerosity that an individual is capable of detecting.

The acuity of the ANS increases with the child’s age, rapidly at first and then more slowly. In six-month-old infants, a reaction to novelty does not occur unless the ratio between the two numerosities to be discriminated is about 1:2 (on average). For example, an infant habituated to the numerosity of sets of eight points exhibits a novelty reaction during the test phase only for numerosities of at least 16 points. This ratio is about 2:3 at nine months (8 can thus be discriminated from 12) and continues to decrease exponentially until it stabilizes at about 7:8 in adulthood. Note, however, that at any given age, stable individual differences in ANS acuity exist, even at the early age of 6 months [22]. Among 14-year-olds, for example, the dispersion of this ratio ranges from 2:3 to 9:10 [20]. The search for correlations between ANS acuity and mathematical ability—a question we will address below—is based on these individual differences.

In sum, the behavioral signature of the ANS in numerosity quantification tasks is the same in humans and some animals. This numerical information processing system also activates homologous brain regions in man and primates. It is operational soon after birth in humans (at three months in [23]). These three characteristics suggest that the ANS was integrated into the genetic heritage of our species relatively early in the course of its phylogenesis and can therefore be seen as the initial state in the development of the numerical quantification function of human beings [24].

### 3.2. The Parallel Individuation System (PIS)

When the number of objects is less than or equal to three, quantification behavior differs from that described above for larger numerosities. Discrimination accuracy no longer depends on the ratio of the two perceived quantities; it is the same for 1 vs. 3, 1 vs. 2, and 2 vs. 3. At a more general level, neither accuracy nor response time depends on the ratio of the two quantities compared. This time, we are dealing with a form of exact representation of small quantities. The system relies on parallel processing in which each object perceived is individualized and represented in short-term memory (STM) by its own symbol, a kind of place holder. The STM representation incorporates the characteristics (shape, area, etc.) that enable the perceiver to track each object through time and space. Unlike with ANS, these continuous variables cannot be dissociated from the number in this system. It is sometimes called the Object Tracking System [18] and sometimes the Parallel Individuation System [25].

The information contained in the PIS-based representation of objects is not itself numerical, but number is implicitly taken into account in the term-by-term comparison between the objects perceived and their symbols already stored in short-term memory. Accordingly, the infants in Wynn’s study [26] showed surprise when one of the objects was secretly added to or taken away from the set initially presented. For up to three objects, this system thus gives an exact representation of quantity and furnishes a non-numerical equivalent of adding or subtracting one object.

The cortical network supporting the PIS is different from that of the ANS. This time, rather than the inferior intraparietal sulcus, it is the occipital-temporal sites that are activated [27]. Like ANS, PIS is operational shortly after birth and develops rapidly. The number of objects an infant can simultaneously take into account goes from one at about one month, to two or three at about 12 months [18]. PIS is also present in the repertoire of primates [28] and like ANS, gives rise to substantial individual differences [29].

The above findings suggest that the quantification of small numerosities (from 1 to 3) is based on a system different from the ANS, but one that, like ANS, became part of the genetic heritage of humanity relatively early in phylogenesis. Some authors call the ANS and PIS “core systems”, defined as domain-specific representational systems that constrain the cultural acquisition of new representations [17,30].

These core systems are very different from what will later become the cognitive structures underlying the concept of number, so these structures are neither innate nor constructed de novo by a general process of equilibration^3^. The resemblance between the quantification behavior of infants and older children lies in the function common to the two kinds of cognitive structures implicated in these behaviors. The development of numerical cognition in children, then, should be regarded as a process that reconstructs the primitive function of discrete-object quantification. The reconstruction process relies on its evolutionary precursors (ANS and PIS), on cognitive abilities that have emerged more recently in the phylogenesis of the human species, and on the knowledge that these abilities have allowed us to construct and transmit. It follows from all this that the reconstruction of the quantification of discrete objects is in fact grounded in a plurality of cognitive processes that treat different kinds of information about number.

## 4. Plurality of Processes Supporting Reconstruction

For Piaget, the construction of numbers in children relied on logical operations, or more specifically, on the synthesis of the operations needed to understand its two great properties: seriation operations, which enable the child to grasp the order relations that structure the sequence of numbers, and class inclusion operations, which enable the child to understand inclusion relationships between sets whose cardinals correspond to consecutive numbers [32]. Later studies painted a much more complicated picture by providing evidence of several other processes that play a substantial role in the construction process. Below is a brief description of what appear to be the most important processes. They can be divided into two main categories on the basis of whether they can be called “analogical”^4^ or “symbolic”. The former quantify on the basis of an analogical relationship between the size of a set of objects and the representation of that size. The latter rely on arbitrary symbols (number words or Arabic numerals) to represent numbers.

### 4.1. Analogical Processing

Two of these processing systems, the ANS and the PIS, were presented above. The analogical nature of ANS lies in the (logarithmic) relation between numerosity and its representation; for PIS, it lies in the correspondence between the number of objects perceived and the number of place holders representing that number in short-term memory. Three other analogical ways of quantifying sets of discrete objects are briefly described below.

#### 4.1.1. One-to-One Correspondence

Before using any numerical symbols, children can judge the equality or inequality of two collections of objects by setting up a one-to-one correspondence between the items in one collection and those in the other. Here, the analogy resides in the spatial correspondence of the collections being compared. This is one of the procedures that Piaget and Szeminska [32] used to test for the conservation of numbers by getting the child to agree on the numerical equality of two rows of tokens before changing one of them. Piaget did not, however, grant this procedure an important role in the construction of numbers precisely because, before the age of six or seven, the equality it permits is not conserved when the spatial spread of the two collections is changed. Yet when children become capable, at the age of about two or three years, of mapping the elements of two collections, they are abstracting an identity relation that paves the way to the notion of exact equality.^5^ This was demonstrated in Mix, Moore, and Holcomb’s experiment [34], where three-year-old children’s ability to judge numerical equivalency (assessed by showing them two cards and asking them to choose which one had the same number of objects as the target card) improved considerably when the children were first given toys designed to stimulate a one-to-one mapping activity (e.g., objects made up of two parts that fit together).

#### 4.1.2. Early Finger Counting

Another way to use one-to-one correspondence to determine how many elements there are in a set is to put up as many fingers as there are elements in the set; this is a peculiar procedure, however, in that it can be regarded as an instance of embodied cognition because the fingers are part of the body [35]. The finger-based representation of the number of objects also rests on an analogy, i.e., it uses as many fingers as there are objects. It implicitly involves several properties of numbers, which is an aid to understanding them later on. The order relation is intrinsic to the sequence of fingers held up [36,37], and so is the successor function:^6^ the same unit—a finger—separates each element from its successor.

There are good reasons, then, to contend that the representation of numbers with fingers contributes to the development of numerical cognition. This was shown in an experiment in which four-year-old children who had not yet learned the concept of cardinality had to state the number of objects displayed on cards (“What’s on this card?” task). In one of the experimental conditions, the children had to reply with a number word; in the other, they had to put up the corresponding number of fingers. The results indicated that response accuracy (measured by how close the response was to the correct answer) was greater in the gestural modality, whether the number was small (1 to 4) or large (5 to 10). Moreover, in cases where both response modalities were used at the same time by the child, if the two responses did not match, the finger response was the more accurate one. As the authors stated, “These results show that children convey numerical information in gesture that they cannot convey in speech and raise the possibility that number gestures play a functional role in children’s development of number concepts” ([38] p. 14).

#### 4.1.3. The Sequence of Number Words

Between the ages of two and three, children learn from people in their surroundings to recite the list of the first few number words. At this stage, the list is merely an unbreakable string of sounds, recited by heart [39]. The words that compose the sound string start becoming separate entities when the child learns to imitate the procedure consisting of saying each word while pointing to a different object. At this point, even if the number words are individualized, they do not yet have the properties of numerical symbols. However, the order in which they are uttered, which is intrinsic to the numerical sequence recited in the past, is analogous to the order relation that structures numbers, and as such can promote its acquisition and comprehension.

### 4.2. Symbolic Processing

Symbolic processing relies on arbitrary symbols (e.g., number words, Arabic numerals) to represent exact numbers of objects. It enables us to directly perform mental quantification operations on these symbols. Unlike the analogical type of processing considered above, it is not based on any kind of resemblance between the symbol and the quantity it represents. The advantage of this route is that the relationships between the symbols are only those defined by the formal rules governing the numerical system. The disadvantage is that it is difficult for children to mentally represent the exact quantity that corresponds, by convention, to each symbol and makes it meaningful.

#### 4.2.1. Subitizing

Subitizing is the rapid apprehension, without counting, of small numbers of objects, from 1 to 3 and sometimes 4. It is very similar to the parallel individuation process presented above and is probably an extension of it [40]. The essential difference is that this kind of rapid apprehension of number is accompanied by verbalization of the corresponding number word (the cardinal of the collection), which requires minimal access to language. This explains why subitizing is rarely observed in children under two. It supplies no information about the ordinal properties of numbers but constitutes an initial form of mapping between a number word and the cardinal of the perceived set.

#### 4.2.2. Counting

Counting is a complex process whose role was underestimated by Piaget but reevaluated by Greco [41]. We are indebted to Gelman and Gallistel [42] for having brought back into the foreground the role of counting in the genesis of the notion of number. These authors identified five principles that must be obeyed to count correctly. The most important ones are one-to-one correspondence, stable order, and cardinality. The principle of one-to-one correspondence states that the items to be counted must be mapped, via a one-to-one correspondence, to the set of number tags used to count (e.g., the set of number words). The stable order principle states that the number tags have a fixed order. The cardinality principle states that the last number used in a count represents the cardinality of the items counted. In the spirit of the neo-nativist era of the seventies, Gelman and Gallistel assumed that these principles were rooted in an innate cognitive structure specific to number. Later research, however, did not confirm their innateness. Rather, they are acquired gradually in the course of early childhood and hardly ever show up in counting behavior before the age of four years [39,43].

The principle of cardinality is assessed using the “Give me N” task (Wynn, [43,44]). The experimenter places the child in front of a set of objects and asks the child to give him/her N objects. Cardinality is considered to be acquired when the child starts to count and, upon arriving at the number N (and only at that moment), gives the experimenter the set of objects just counted. Understanding that the cardinal of a collection also and necessarily includes all the cardinals of smaller collections is a much more advanced stage that no doubt requires mastery of logical operations such as class inclusion, to which Piaget granted a unique role. Children who master the principle of cardinality have begun to understand that each of the number words in the sequence they know refers to an exact quantity that is specific to it.

The various processes reviewed above, both analogical and symbolic, have a shared function, that of quantification, but they perform it by processing different information. Plurality of processing opens up the possibility of interactions, a potential source of development [45].

## 5. Interaction

How are these different processes related to each other in the course of development? Are they independent or interdependent? If they are interdependent, are the relationships between them symmetrical or asymmetrical? If they interact, what types of interactions are involved, conflicting ones or mutually supportive ones?

These questions will be addressed below for the two processing systems reviewed here, the analogical system of approximate number representation or ANS, and the symbolic system of exact representation. Both are subject to developmental change over time: ANS acuity increases with age and so do numerical skills. Both give rise to large individual differences, that is, ANS-acuity differences and performance differences in initial numerical skills (e.g., number list, counting, cardinality, small-number operations). Within the past few years, many experiments have looked at these individual differences, generally using correlation methods, to determine how the two systems are related. I will begin with an overview of the results of studies demonstrating the impact of the ANS on the symbolic system. Then I will present the results of studies showing the opposite impact.

### 5.1. Does ANS Have an Effect on the Development of the Symbolic System?

It is not possible here to describe all of the studies on how the ANS affects numerical-skill development, but the reader will find reviews of this question in Mussolin et al. [46] and in two meta-analyses (Fazio et al. [47]; Chen and Li, [48]). The conclusions of these studies are convergent, so to summarize the results, I will rely solely on the Chen and Li [48] meta-analysis (which is the most comprehensive).

The cross-sectional studies meta-analyzed dealt with 31 studies involving 36 independent samples. Many factors differed across experiments, including the age of the participants, the covariables controlled, the tasks used to assess ANS acuity, the indexes calculated to establish its signature, and the tasks employed to assess mathematical abilities. A positive correlation was found in 35 of the 36 samples and was significant in 20 of them. The mean correlation, 0.24, was not very high but significant. None of the factors just mentioned had a significant impact on the magnitude of the correlation.

The existence in the cross-sectional studies of a correlation between ANS and numerical skills does not, however, tell us anything about the direction of the relationship between the two variables. Longitudinal studies conducted to find out whether individual differences in ANS acuity at time *t* predict performance differences in numerical skills at time *t* + 1 are better suited to determining the direction of the relationship. Eight longitudinal studies involving 11 samples were included in the Chen and Li [48] meta-analysis. Once the covariables measuring more general cognitive abilities were controlled, the mean correlation between ANS acuity and numerical-skill performance was 0.25, which is similar in magnitude to that found in the cross-sectional research.

The structural analysis of the relationships between the variables provides further information. Chu et al. [49] showed that among the children they examined at the age of four and then again at the end of the school year, ANS acuity at four years predicted numerical skills at the end of the year. However, this relationship was fully mediated by the relationship between performance differences on the cardinality task and differences in numerical skill level. Insofar as the ANS precedes cardinality, this result suggests that differences in ANS acuity are the source of differences in the acquisition of cardinality, which, in turn, contribute to differences in numerical skills.

Other convincing data on the direction of the relationship between the ANS and symbolic arithmetic can be found in training experiments. Hyde et al. [50] gave first graders two training sessions on non-symbolic numerical approximation (approximate addition of sets of points or approximate comparison of numbers). The results showed that in both cases, the children’s performance on a test of exact symbolic arithmetic was significantly better than that of the children in the control groups. Similar results were found by Obersteiner et al. [51]. Park and Brannon [52], who studied adult participants, also showed that training in approximate addition and subtraction of sets of points raised both ANS acuity and performance on a test of exact addition and subtraction.

It thus seems reasonable to conclude that the system of approximate number representation affects the development of the symbolic system of exact representation.

### 5.2. Does Learning the Symbolic System Have an Effect on ANS Acuity?

The correlation found in the cross-sectional studies analyzed above could also be due to an effect of numerical-skill acquisition on ANS acuity, but it does not demonstrate this effect.

Here again, longitudinal studies offer less ambiguous information. In Chen and Li’s [48] meta-analysis, four longitudinal studies (with five samples) tested the relationship in this direction. The mean correlation was 0.23, which is comparable to the value found in the longitudinal studies where the relationship was tested in the other direction. Here again also, an analysis of the structure of the relationships at play can supply more precise information. Mussolin et al. [53] assessed numerical skills and ANS acuity in children at the age of 4 years and then again seven months later. Using the cross-correlation method, they showed that numerical skills at 4 years predicted ANS acuity at the end of the school year, but not the opposite.

Other convincing data on the relationship in this direction can be found in experiments analyzing the effects of numerical-system learning on ANS acuity. Opfer and Siegler [54] studied the developmental time course of the function linking the representation of the magnitude of numbers to their real magnitude. Children of different grades in school were asked to position numbers from different numerical intervals (0 to 10, 10 to 100, 100 to 1000, etc.) on a continuous line bounded on both ends. Their results showed that the logarithmic function became a linear function as school grade increased, but only interval by interval. For example, for the interval 0–100, the function was logarithmic for the preschoolers and linear for the second graders, but for these same second graders, the function was still logarithmic for the interval 0–1000, and so on. Hence, learning the rule of succession—whereby each number leads to the next by iteration of one unit and all intervals between two consecutive numbers are equal—is not transferred in an immediate way to the approximate representation of all numbers. It is more likely that the experience acquired through manipulation of the numbers in the interval being learned in each school grade transforms the ANS-based representation of approximate magnitudes.

Cross-cultural studies shed another type of light on this issue. Piazza et al. [55] studied the approximate estimation of numerosities in a native Amazon population, the Mundurucù. The language of these people has a very limited lexicon of number words and they have no symbolic way whatsoever to process discrete quantities. They can, however, compare the numerosities of two sets, or estimate their approximate sum. The authors compared adults who had gone to school to those who had not, on a task involving the approximate estimation of numerosities. The results showed that for all participants, schooled or not, the function that linked the discrimination capacity to the size of the compared numerosities looked very much like the logarithmic function already found in industrialized nations. However, Weber’s ratio was significantly lower—indicating greater acuity—among the Mundurucù who had been taught arithmetic, and acuity grew as the number of years of schooling rose.

It thus seems reasonable, here also, to conclude that learning the symbolic system affects the developmental time course of the ANS, in particular by improving its acuity and changing the shape (from logarithmic to linear) of the function that links approximate representations of magnitude to their real magnitudes.

If each process that performs the quantification function has an impact on the unfolding of each of the others, then the relationship between them is one of mutual support, and together they form a dynamic system capable of self-organization [15]. Furthermore, whenever several processes fulfill the same function, they can also be related by substitution, i.e., substitution of one for another, depending on the situation and the individual.

## 6. Substitution and Variabilities

In situations that call upon the quantification function, it is hypothesized that the various processes capable of fulfilling that function are competing with each other. Depending on the type of information they process, their activation probabilities—or their respective weights if they are activated in parallel—are likely to vary as a function of the situation and the individual. The resulting possibilities for substitution are assumed to be one of the sources of within- and between-individual variability. This section gives three examples of variability that can be explained in terms of substitution (when all co-functional processes are available) or compensation (when one of those processes is impaired). The first example is drawn from the typical development of numerical cognition, the second from atypical development, and the third from arithmetic problem solving.

### 6.1. An Example of Substitution Relationships in Typical Development

Acquiring the principle of cardinality is decisive in the acquisition of the numerical system. It is at this time, between the ages of three and a half and four and a half that children begin to understand that each of the numerical symbols they know (number words, digits) represents an exact quantity, one that is specific to it. To grasp this, children must map these symbols to the analogical representations of quantity they have at their disposal. By the time they are two and a half, on average, children know that number words refer to quantities, but while they know and can learn to recite these words, they do not know the correspondence between the words and the quantities. First, they learn the meaning of “1”, but it will take them several months to learn the meaning of “2”, and then several additional months to learn the meaning of “3”. The discovery of the meaning of numbers larger than 3 or 4 marks the transition to another stage and is based on a different process. This new capacity is a testimony to the fact that the child has discovered the principles of counting, in particular that of cardinality, and has become capable of generalizing those principles to larger numbers. The transition occurs between the ages of three and four, on average, and the criterion is the child’s ability, on the “Give me N” task, to give the exact quantity corresponding to the number requested by the experimenter, for a number greater than four.

#### 6.1.1. Two Hypotheses about the Route to Cardinality

There is an ongoing debate about the pathway taken by children to arrive at the principle of cardinality. For some authors (e.g., Le Corre, Carey, [25]; Carey et al. [56]), the transition rests on the “parallel individuation system” (PIS), the only one capable of enabling the child to map the number words from “one” to “three” or “four” to the exact quantities to which they correspond. For others (Dehaene [24]; Feigenson et al. [17]; Piazza [18]; Wynn [44]), the mapping can be achieved via the approximate number system (ANS).

##### The ANS-to-Word Pathway

Authors who advocate the role of the ANS in the acquisition of cardinality believe that this approximate representation system is sufficient to account for the increasingly precise matching between magnitudes and numerical symbols. They hypothesize that children begin mapping the first few number words because they are the most frequent in the language. Moreover, the quantities corresponding to these numbers are related to each other in ways that make them discriminable by three-year-olds. At this age, children are capable of discriminating ratios of 3:4, so they must also be able to discriminate 1 from 2, 2 from 3, and all the more so 1 from 3, since the ratios of these comparisons (1:2, 2:3, and 1:3) are easier than 3:4. Moving up from these small numbers to the following ones can be achieved by realizing that going from one number to the next involves adding one object.

##### The PIS-to-Word Pathway

Carey and her colleagues of course agree that numerical symbols are mapped to approximate representations of their magnitude, but they do not agree that this is what leads to cardinality [25,56]. Their hypothesis is that this type of mapping can only take place once the principle of cardinality has been acquired. One of the reasons for their reluctance is that cardinality is based on an exact correspondence between the quantity and the symbol that represents it, yet mapping via the ANS only supplies approximate representations. On the other hand, as we have seen above, PIS gives the exact representation outright for the numbers from 1 to 3 or 4. The parallel individuation of the elements of a small set nevertheless only furnishes fleeting representations in short-term memory, whereas representations of the magnitudes of number words must be permanently stored in long-term memory. To account for the fact that the mapping done in short-term memory is transferred to long-term memory, Carey and colleagues hypothesize that the representation of a set is enriched by knowledge of other sets of the same size (e.g., “me” for 1, “Mommy and Daddy” for 2, “Mommy, Daddy, and me” for 3. This process is called the “enriched parallel individuation system”. It is assumed to enable mapping of each of the first three or four numbers to an exact representation of the quantities associated with them. When going from the exact representation of the number 1 to that of the number 2, and likewise for 2 to 3, the child has the opportunity, here also, to grasp that going from a given number word to the next involves adding 1. Cardinality, discovered on small numbers, thanks to PIS, would then be generalized to become the cardinality principle that counting obeys.

#### 6.1.2. Experiments Aimed at Choosing between These Two Hypotheses

Experiments devised to decide which is the better hypothesis about the route to cardinality have given rise to contradictory results. The contradictions result from unexpected within- and between-individual variations in the execution of tasks where a number must be matched to a quantity of objects, or a quantity of objects must be matched to a number. This section begins by summarizing the findings, and then looks at how the model of substitution might help resolve the contradictions.

The first experiment aimed at choosing the better hypothesis was conducted by Le Corre and Carey [25]. It was designed around two main ideas. The first was that if the pathway to cardinality rests on ANS, we should find its signature. The second was that approximate ANS-based mapping most certainly exists for large numbers, but it should only be found after the principle of cardinality has been acquired.

In this study, Le Corre and Carey tested children whose mean age was 3.11 on two tasks, one (“Give me N”) designed to detect their knowledge of the cardinality of numbers, the other (“Fast Cards”) designed to see whether the signature of the ANS would be found in the children’s matching behavior. In “Give me N”, those who responded correctly only for N = 1 were labelled 1-knowers, and so on up to 4-knowers. Those who responded correctly only for these small numbers (1 to 4) were put in a group called “subset-knowers”. Those who responded correctly for larger numbers, at least up to 5 or 6, were put in a group called “cardinality-principle knowers” (CP-knowers). In “Fast Cards”, the children had to say the number word corresponding to the number of elements (circles) on the card shown by the experimenter. The number of circles varied between 1 and 10, but only the responses given for the larger numbers (5 to 10) were analyzed. To prevent the children from counting the circles, each card was shown for only one second.

As hypothesized, the ANS signature (logarithmic function) in the “Fast Cards” task was found only among the CP-knowers. According to the authors, the fact that the signature of the ANS was not found among the subset-knowers but only among the CP-knowers shows that the pathway toward cardinality does not rely on the ANS. It was only after the principle of cardinality had been acquired that mapping of large numbers to an approximate representation of their magnitude was observed.

In a more recent experiment, Wagner and Johnson [57] also studied the matching of number words to the magnitudes of sets of objects, by children whose mean age was 4.1 on the “Give me N” task. But unlike Le Corre and Carey, they did not stop task execution after the last correct response but went up to the number 10 for all children. The results showed that the mean number of objects given by the child increased with the magnitude of the difference between the number requested by the experimenter and the knower level of his/her last correct answer. The standard deviation also increased and the coefficient of variation (standard deviation divided by the mean) was constant, which is the signature of the ANS. The authors concluded that ANS plays a role in the representation of the magnitude of number words for children who have not yet acquired the principle of cardinality. This is the opposite of what Le Corre and Carey found. It should be noted, however, that the tasks used were not the same. “Fast Cards” consists of presenting a set of items and asking the child to say the number word that corresponds to it, whereas “Give me N” consists of saying a number word and asking the child to give the corresponding number of items. The direction of the required mapping is thus quantity to number word in the former case, and number word to quantity in the latter. Note also that the two tasks were not performed by the same children.

In an attempt to shed light on the contradictory results of the above studies, Odic et al. [58] had children (mean age 3.6) perform both mapping tasks: mapping in the quantity-to-word direction (“Fast Cards”) and mapping in the word-to-quantity direction (an adapted version of the “Give me N” task). The results of the “Fast Cards” experiment replicated Le Corre and Carey’s findings: in the quantity-to-word direction, the function linking the magnitude of the set of points to the magnitude of the number word produced had a positive slope only for some of the CP-knowers. The results of the adapted version of the “Give me N” task, in which the mapping was in the word-to-quantity direction, replicated Wagner and Johnson’s results: the slope of the function linking the number of items to the number word requested by the experimenter was positive and significantly different from zero; this was true not only for the CP-knowers but also for the 2-knowers and the 3-knowers. These results allowed the authors to conclude that “before children have become CP-knowers, they are able to map from a discrete number word representation, e.g., 10, to a region on the continuous ANS mental number line” (p. 118).

Since the same children did both matching tasks here, there was within-individual variation in performance according to the mapping direction: quantity-to-word or word-to-quantity. The authors interpreted this within-individual variation as being due to a difference in difficulty comparable to that found in language development between production tasks (here production of a number word in the quantity-to-word direction) and comprehension tasks (here comprehension of a number word in the word -to-quantity direction), production being more difficult than comprehension.

However, in another experiment where both tasks were given to the same children, there was also within-individual variation in mapping success, but it went in the opposite direction (Gunderson et al. [59], experiment 2). In that experiment indeed, the children’s responses exhibited the ANS signature in the quantity-to-word direction (“Fast Dots Task”) but not in the “word-to-quantity” direction (“Give me N” Task). If the observed within-individual variations between these two tasks were only differences in difficulty, we would need to explain why the task that was the easiest for the children in Odic et al. experiment was the most difficult for the participants of Gunderson et al. experiment.

We are thus faced with a case of *within-individual* variation that went in the opposite direction for different children. This *between-individual* variation was probably due to the way in which the subjects in Gunderson et al. experiment 2 were selected. The authors chose older subset-knowers than in their experiment 1 in order to observe the behavior of subset-knowers whose ANS development was more advanced. To find children who were older that those in experiment 1, but still had not acquired the cardinality principle, they recruited participants (mean age 4.2, range 3.11–5.5) from nursery schools in a neighborhood with a lower sociocultural level than in their experiment 1.

#### 6.1.3. A Possible Interpretation of the Observed Variabilities

The children just mentioned were all subset-knowers, that is to say, children able to match at least some small numbers (from 1 to 4) to the exact quantities that correspond to them, which is the signature of the PIS. Nevertheless, contrary to what Carey and Le Corre thought they were, at the same time, able-under certain conditions–to form an approximate representation of the magnitude corresponding to numbers greater than 4, which is the signature of the ANS. Therefore, in these children who have not yet acquired the principle of cardinality, both of these quantification processes are available for matching the number words to a representation of their magnitude.

As a consequence, subset-knowers have the opportunity to substitute the ANS for the PIS in the course of a mapping task when the latter becomes ineffective, but this possibility seems to depend on two conditions: the characteristics of the subjects and the direction of the mapping. Concerning the characteristics of the subjects, the age difference between those included in Gunderson et al. experiment and those included in Odic et al. experiment is confounded with a difference in sociocultural level. Children with a low sociocultural level are known, on average, to be less successful in the verbal domain than in the spatial domain and vice versa for those with a high sociocultural level. Concerning the direction of the mapping, the spatial configuration of the set of objects is the prime in the quantity-to-word direction, whereas the number word is the prime in the word-to-quantity direction. Therefore, the within- and between-individual variations observed in the results of the mapping tasks examined above could be due to the fact that the ability to substitute the ANS for the PIS when the number of objects goes up depends on the situation (here, the direction of the mapping), the characteristics of the individual (here his/her skill level in the verbal and spatial domains), and the potential interaction between the two.

Insofar as these experiments were not designed to test for substitution relationships, their interpretation can only be hypothetical. But these hypotheses could be tested by having children carry out tasks that assess verbal and spatial aptitudes independently.

### 6.2. An Example of Compensation in Atypical Development

Whenever a deficiency, whether genetic or accidental, is such that one of the co-functioning processes is absent from the child’s repertoire or is inefficient, the existence of substitution relationships enables another process to take its place, either partially or totally. The word *compensation* is more suitable in this case than the word substitution, because a substitution relationship is reciprocal. We find compensation relationships in certain kinds of atypical development.

Children with Williams Syndrome, for example, have a unique developmental profile in which verbal abilities are less impaired, relatively speaking, than spatial abilities. Their acquisition of arithmetic is delayed. Ansari et al. [60] wondered whether these children could acquire numerical skills via the same route as typically developing children. They focused in particular on the acquisition of the principle of cardinality.

They studied two groups of children matched on spatial abilities: a group of typically developing children, mean age 3.5, and a group of Williams Syndrome children, mean age 7.6. Both groups had taken verbal aptitude tests. The cardinality principle was in the process of being acquired in each group, with comparable mean performance levels and dispersions. The most interesting finding is that for the typically developing group, individual differences in the scores on the cardinality task “Give me N” were linked to individual differences in spatial aptitude, whereas in the Williams group, the cardinality scores were linked to differences in verbal aptitude. This suggests that the two groups follow different pathways to arrive at the principle of cardinality.

Van Herwegen et al. [61] later study sheds an interesting light on this issue. These authors had a group of nine Williams children (mean age 2.11) perform a task involving the approximate estimation of large numerosities, and a task involving the exact discrimination of small numerosities. The children were capable of exactly discriminating the small numerosities but were unable to discriminate the large numerosities. This result suggests that the ANS, known to be operational shortly after birth in typically developing children, has not yet been acquired by Williams children who are nearly three years old. Insofar as the mental number line of the ANS has spatial properties [62], it could very well be that the ANS deficit of these children is linked to their spatial impairment. The PIS, where words are mapped with exact quantities, may not be affected as much because these children’s language abilities are less altered than their spatial ones. The pathway taken by Ansari et al.’s children with Williams Syndrome, who, in spite of their impairment, were able to construct the notion of cardinality, is thus likely to be a route that relies on their relatively well-spared abilities, namely, the PIS and language.

Why, then, are Williams children so far behind in learning the principle of cardinality, knowing that the PIS is available in their repertoire? Undoubtedly, it is because this system can only partially compensate for ANS deficits. If this is indeed the case, then the above finding points in the same direction as those analyzed in the preceding section. It shows—a contrario—that typical development relies on both systems, including in the phase preceding the acquisition of cardinality.

Deaf individuals who lack a conventional language (spoken or signed) exhibit the opposite deficit. These individuals, called homesigners, devise their own gestures to communicate. Their gestures are not used as a tally system, and homesigners do not have the equivalent of a counting list or a counting routine. When they communicate about the magnitude of sets of objects, they are accurate for sets from 1 to 3. For sets from 4 to 20, they are approximately, but not exactly, correct. They understand that each set has an exact numerical value, but they do not have an errorless way of arriving at a gestural representation of that value. Their responses are centered on the target value, with a small dispersion around it [63]. These facts confirm the specific role of the PIS in the exact representation of the numbers from 1 to 3. They also show that acquisition of the cardinality principle is not a necessary condition for the development of the ANS. The system of approximate representation can partly compensate for impairment in conventional (spoken or signed) language, but it is also clear that both systems are necessary for optimal efficiency of the quantification function.

### 6.3. An Example of Substitution in Arithmetic Problem Solving

Within- and between-individual variations corresponding to pathway differences in long-term numerical development also exist in short-term arithmetic problem solving, in the form of variations in strategies. Siegler proposed a selectionist model of the developmental course of strategies, inspired by the conceptual framework of Darwin’s theory of evolution, in particular its concepts of variation and selection [7]. This theoretical framework was applied to the development of arithmetic knowledge in a microgenetic study where the solving strategies applied to small-number addition problems were analyzed in five-year-old children, [64]. No fewer than eight different strategies were identified. This analysis revealed not only the existence of strong between-individual variability—indicating that the dominant strategy was not the same for all children—but also of strong within-individual variability. No matter what their dominant strategy was, all of the children also used other strategies at one time or another. Their choice sometimes depended upon the characteristics of the problem (for example, the size of the addenda or to the order in which they were stated), and sometimes on the point in time (when the same problem had to be solved again). This within-individual variability plays an important role in development. In Siegler’s selectionist approach, it is considered a necessary condition for the selection process via which the most efficient and least costly strategy for this particular subject is gradually adopted for this particular category of problems, at this particular point in time. Its role is also important in the development of new strategies by the way of which elements are taken from strategies whose within-individual variability has enabled exploration.

There are both similarities and dissimilarities between Siegler’s conceptual framework and that of the reconstructivist approach I am proposing here. The similarities lie in the emphasis put on the plurality of processes and on the relationships of substitution (between strategies or between processes) used to account for variability. The dissimilarities lie in the time scale of the processes observed and in the developmental mechanism brought to bear. Concerning the time scale, the strategies take effect in the short term of problem-solving (seconds or minutes) whereas reconstruction occurs in the long term (months or years). Concerning the developmental mechanism of change, Siegler’s theory favors the variation-selection pair, inspired by the theory of evolution, while the approach I am proposing here, inspired by dynamic-system theories, favors mutual support as a source of self-organization. From my point of view, Siegler’s theory is effective at explaining the changes that takes place among a set of strategies—or components of strategies—already existing in the child’s repertoire, but is less convincing to explain the emergence of truly new strategies.

## 7. Discussion

The concept of numbers is not constructed solely by the coordination of logical operations, as Piaget thought [32]. Nor does it result from the actualization of an innate cognitive structure that harbors the principles of counting, as Gelman and Gallistel thought [42]. These two theories, one constructivist, the other nativist, made major contributions to furthering our knowledge of the genesis of numbers in children, but later work has pointed out their limitations. The cognitive processes underlying numerical development are in fact more numerous and more diverse. Some rely on an analogical representation of quantity, others on a symbolic representation. Some make use of language, others do not. Some are best suited to representing small quantities, others to representing large numerosities. Some supply an approximate representation of quantity, others an exact representation. Although these diverse cognitive processes treat different information, they perform a common function (hence the term “co-functional”)—namely, the quantification of sets of discrete objects.

Two of them, the ANS and the PIS, have a status of their own due to their anteriority on the phylogenetic and ontogenetic levels. We know very little about the exact role played by each of these core systems in the orchestration of the processes that take effect later in the development of numerical cognition. We can nevertheless assume that in situations that call upon the quantification function, all processes that have affordances in these situations are activated. At the beginning of child development, this only concerns the core systems, but as soon as other processes become operational, a growing number of co-functional processes are activated in quantification situations. During this phase—and by virtue of their anteriority—the neural structures that have been supporting the function’s primitive form would attract those that are co-activated at the same time by quantification tasks. The system gradually formed in this manner is not built through the coordination of isolated, initially unrelated schemes, as Piaget thought. Rather, it is formed by the integration of new cognitive capacities that serve a preexisting function, around which they themselves are structured at the same time as they are transforming and reconstructing the function.

Two phases must be distinguished in this reconstruction process: invention and learning. During the initial invention phase, human beings gradually discovered new possibilities for quantification, ones that were more precise and more efficient, opened up by the evolution of the brain in their species. Some examples of inventions, for instance, are when a human being got the idea to have a stone, a finger, a notch, or a stick correspond to each member of his herd, or imagined assigning a name or a gesture to an exact quantity, etc. This phase of the reconstruction process took thousands of years and continues today. It gave rise to the number-based culture in which the children whose development we are now studying are immersed. The second form of reconstruction is not void of inventions—which for children are rediscoveries—but relies much more heavily on learning, and for this reason, takes only a few years. In a cultural environment where numerical information is an integral part of daily life, situations confronting children with quantification problems activate the available primitive quantification systems, granted, but they also activate all cognitive processes that can help in understanding the language, gestures, signs, etc. used in those situations by people in their surroundings. The co-activation of all processes that are co-functional but treat different information about quantity has implications not only from the developmental standpoint but also from the point of view of variability.

From the developmental standpoint, any process can affect the unfolding of any of the other co-functional processes, since each one treats information that the others cannot access directly on their own. If this is the case, the interactions between them can take on the form of relations of mutual support, and they form a dynamic system capable of self-organization. This is a source of development that differs from those rooted in the maturation of the nervous system and in learning. The findings on the reciprocal impact of the approximate number system and the symbolic system are compatible with the mutual-support hypothesis. It would be interesting to find out whether this type of interaction can be generalized to all cognitive processes that perform the quantification function.

From the standpoint of variability, a system containing a plurality of co-functional processes also opens up the possibility of substitution relationships. Reuchlin’s [16] model of substitution can account for the contradictions between the results of experiments designed to show that only one of two co-functional processing systems, here ANS or PIS, is at play in the child’s pathway toward cardinality. Substitution relationships can give rise to two sorts of variability in behavior. Firstly, we have within-individual variability, which is situation-dependent and stems from the fact that a given co-functional process does not necessarily have the same affordance for all situations (note in passing that situation-dependent alternation between the various processes promotes their interaction). Secondly, we have between-individual variability, which stems from the fact that in a given situation, not all individuals necessarily select the same process among those available in their repertoire, to perform the function in question. These different forms of variability are manifestations of the plasticity engendered by the existence of multiple co-functional processes. Variability, here, is not seen as a peculiarity that must be neutralized in order to gain access to the general laws of cognitive functioning and development, but as a consequence of those laws. It follows that studying the different forms of variability opens up an original window on the study of the general laws of cognitive functioning and development.

The reconstructivist approach illustrated here using the example of quantification could be generalized to other major cognitive functions that have also been integrated–into primitive forms–in the genetic heritage of the human species. Categorization, for example, also has primitive forms that are operational shortly after birth [65]. In addition, there are a number of co-functional processes involved in the developmental reconstruction of this function that occur in a more reliable, more efficient, and more controlled form. At the most general level, we find both similarity-based and theory-based processes. At a more specific level, several co-functional processes are available: taxonomic categorization (both birds and crocodiles are animals), thematic categorization (birds live in nests), “slot-filler” categorization (both oatmeal and bacon are breakfast foods) [66]. It would therefore be interesting to see if, in this domain as well, these co-functional processes exhibit mutual support relationships able to trigger self-organization of the categorization system, as well as substitution relationships able to explain the within- and between-individual variabilities observed in categorization tasks.

To look for development through the window of variability, one must adopt a different methodological approach from that most frequently used in developmental research. Rather than examining each co-functional process separately, one needs to study the dynamics of the system they form. This requires an idiographic [67] and longitudinal study of any within-individual variation in the developmental level attained by each child in each co-functional process. This would allow us to study the stability of each child’s within-individual variation profile over time, and also to study between-individual variations in these within-child variations.

To my knowledge, studies that have taken this approach to numerical cognition are rare. One such study was transversal only. Dowker [68] evaluated the developmental level of four-year-olds in some of the skills involved in the construction of number, including counting, cardinality principle, order irrelevance principle, repeated addition of 1, and repeated subtraction of 1. A significant but moderate link was found between the performance on these different components, none of which seemed to be a prerequisite for the others. For example, 22% of those who mastered counting had not mastered cardinality and 41% of those who were poor counters had mastered cardinality. Another such study was longitudinal [69]. In this case, the authors made three assessments (at ages 4, 5, and 6) of the developmental level reached in three kinds of skills (non-symbolic, mapping, and symbolic). A confirmatory factor analysis of the data showed that the three types of skills were saturated by three distinct factors at ages four and five, but by a single common factor at age six. This means that there were between-individual differences in the direction of the within-individual variations on these different skills at ages four and five, but not at age six. In other words, the pathway differences observed at four and five years disappeared at six years. However, in this kind of factorial approach, the individual level is lost [70] and we do not know if the children contributing to each of the three factors were the same at ages four and five. There is one other study that gives us an idea of the degrees of freedom that substitution possibilities leave in the developmental pathway, and also of the variety of methods that can be used to highlight this phenomenon [36]. In this case study, the author described the developmental pathway of a child who learned to count by putting his fingers in a one-to-one correspondence with the objects to be counted and using the finger collection thus constituted as a representation of number. It was not until some time later that the child acquired number words by matching them to the analog representation of quantity that constituted the collections of fingers.

The pathway differences that the reconstructivist approach would uncover would be doubly interesting. Theoretically, these differences will tell us about the degrees of freedom that exist along the developmental pathway leading to the reconstruction of a given cognitive function. Practically, better knowledge of the various possible pathways could help with devising learning methods suited to each route.

## Footnotes

1 In his model, Reuchlin [16] used the French word “*vicariance*” to refer to this type of relationship between processes. For lack of an exact equivalent in English, I use the expression “substitution” to translate this term.

2 Weber’s fraction *w*, which is constant across a range of numerosities, is the difference between the two closest discriminable numerosities, normalized by their size. The same information is given by the coefficient of variation (standard deviation/mean).

3 In Piaget’s theory, Equilibration is a general process of cognitive functioning which, whatever the knowledge domain, regulates: (1) the equilibrium between the *assimilation* of objects into the action schemes of the subject and the *accommodation* of these schemes to the objects; (2) the equilibrium in *reciprocal assimilation and accommodation* between schemes; (3) at the higher level, equilibrium between the *differentiation* of schemes and their *integration* in a more general structure [31].

4 In the literature, these processes are in fact usually called non-symbolic or non-verbal, which emphasizes what they are *not* and advantageously avoids having to make a statement about what they *are*. In my mind, they are analogical, but this is clearly a point of discussion.

5 This is not yet a relation of numerical equality because if, say, the identity is modified by replacing an item in one of the two collections by an item that is not identical to it, the relation of numerical equality is disrupted in the child’s eyes [33].

6 The successor function is a rule establishing the existence of a minimal quantity—one—that corresponds to the minimal distance between two consecutive numbers.

## References

[1] Lautrey J., Mazoyer B., van Geert P. (2002). Invariants et Variabilités dans les Sciences Cognitives.

[2] Thelen E., Smith L.B. (1994). A Dynamic Systems Approach to the Development of Cognition and Action.

[3] Van Geert P.L.C., van Dijk M. (2002). Focus on variability: New tools to study intra-individual variability in developmental data. Infant Behav. Dev..

[4] Van Geert P., Demetriou A., Raftopoulos A. (2004). Dynamic modeling of cognitive development: Time, situatedness and variability. Cognitive Developmental Change: Theories, Models and Measurement.

[5] Bates E., Johnson M.H., Karmiloff-Smith A., Parisi D., Plunkett K. (1996). Rethinking Innateness: A Connectionist Perspective on Development.

[6] Rinaldi N., Karmiloff A. (2017). Intelligence as a developing function: A neuroconstructivist approach. J. Intell..

[7] Siegler R.S. (1996). Emerging Minds.

[8] Lautrey J., de Ribaupierre A., Rieben L. (1985). Intra-individual variability in the development of concrete operations: Relations between logical and infralogical operations. Genet. Soc. Gen. Psychol. Monogr..

[9] Lautrey J., Sternberg R.J., Grigorenko E. (2002). Is there a general factor of cognitive development?. The General Factor of Intelligence: How General Is It?.

[10] Demetriou A., Spanoudis G., Kazi S., Mouyi A., Žebec M.S., Kazali E., Golino H., Bakracevic K., Shayer M. (2017). Developmental differenciation and binding of mental processes with g through the life-span. J. Intell..

[11] Lautrey J., Reuchlin M., Lautrey J., Marendaz C., Ohlmann T. (1990). Esquisse d’un modèle pluraliste du développement cognitif. Cognition: L’universel et L’individuel.

[12] Lautrey J., Case R., Edelstein W. (1993). Structure and variability: A plea for a pluralistic approach to cognitive development. The New Structuralism in Cognitive Development: Theory and Research in Cognitive Development.

[13] Lautrey J., Sternberg R.J., Lautrey J., Lubart T. (2003). A pluralistic approach to cognitive differentiation and development. Models of Intelligence: International Perspectives.

[14] van der Maas H.L., Molenaar P.C. (1992). Stagewise cognitive development: An application of catastroph theory. Psychol. Rev..

[15] Van der Maas H.L.J., Dolan C.V., Grasman R.P.P., Wicherts J.M., Huizenga H.M., Raijmakers M.E.J. (2006). A dynamical model of general intelligence: The positive manifold of intelligence by mutualism. Psychol. Rev..

[16] Reuchlin M. (1978). Processus Vicariants et Différences Individuelles. J. Psychol..

[17] Feigenson L., Dehaene S., Spelke E. (2004). Core systems of number. Trends Cogn. Sci..

[18] Piazza M. (2010). Neurocognitive start-up tools for symbolic number representations. Trends Cogn. Sci..

[19] Xu F., Spelke E.S. (2000). Large number discrimination in 6-month old infants. Cognition.

[20] Halberda J., Mazzocco M.M.M., Feigenson L. (2008). Individual differences in non-verbal acuity correlate with maths achievement. Nature.

[21] Nieder A. (2013). Coding for abstract quantity by number neurons of the primate brain. J. Comp. Physiol. A.

[22] Libertus M.E., Brannon E.M. (2010). Stable individual differences in number discrimination in infancy. Dev. Sci..

[23] Izard V., Dehaene-Lambertz G., Dehaene S. (2008). Distinct cerebral pathways for object identity and number in human infants. PLoS Biol..

[24] Dehaene S. (2011). The Number Sense: How the Mind Creates Mathematics.

[25] Le Corre M., Carey S. (2007). One, two, three, four, nothing more: An investigation of the conceptual sources of the verbal counting principles. Cognition.

[26] Wynn K. (1992). Addition and subtraction by human infants. Nature.

[27] Hyde D.C., Spelke E. (2010). Neural signatures of number processing in human infants: Evidence for two core systems underlying numerical cognition. Dev. Sci..

[28] Hauser M.D., Carey S. (2003). Spontaneous representation of small numbers of objects by rhesus macaques: Examination of content and format. Cogn. Psychol..

[29] Vogel E.K., Machizawa M.G. (2004). Neural activity predicts individual differences in visual working memory capacity. Nature.

[30] Spelke E., Kinzler K. (2007). Core knowledge. Dev. Sci..

[31] Piaget J. (1978). The Development of Thought.

[32] Piaget J., Szeminska A. (1941). La Genèse du Nombre Chez L’enfant.

[33] Izard V., Streri A., Spelke E.S. (2014). Toward exact number: Young children use one-to-one correspondence to measure set identity but not numerical equality. Cogn. Psychol..

[34] Mix K.S., Moore J.A., Holcomb E. (2011). One to one play promotes numerical equivalence concepts. J. Cogn. Dev..

[35] Bender A., Beller J. (2012). Nature and culture of finger counting: Diversity and representational effects of an embodied cognitive tool. Cognition.

[36] Brissiaud R., Bideaud J., Meljac C., Fischer J.-P. (1992). A tool for number construction: Finger symbol sets. Pathways to Number: Children’s Developing Numerical Abilities.

[37] Roesch S., Moeller K. (2015). Considering digits in a current model of numerical development. Front. Hum. Neurosci..

[38] Gunderson E.A., Spaepen E., Gibson D., Goldin-Meadow S., Levine S. (2015). Gesture as a window onto children’s number knowledge. Cognition.

[39] Fuson K.-C. (1988). Children’s Counting and Concepts of Number.

[40] Piazza M., Fumarola A., Chinello A., Melcher D. (2011). Subitizing reflects visuo-spatial object individuation capacity. Cognition.

[41] Gréco P., Gréco P., Morf A. (1962). Quantité et quotité: Nouvelles recherches sur la correspondance terme à terme et la conservation des ensembles. Structures Numériques Elémentaires.

[42] Gelman R., Gallistel C.R. (1978). The Child Understanding of Number.

[43] Wynn K. (1990). Children’s understanding of counting. Cognition.

[44] Wynn K. (1992). Children’s acquisition of number words and the counting system. Cogn. Psychol..

[45] Lautrey J. (2014). Approche pluraliste du développement: L’exemple de la construction du nombre. Enfance.

[46] Mussolin C., Nys J., Leybaert J., Content A. (2016). How approximate and exact number skills are related to each other across development: A review. Dev. Rev..

[47] Fazio L.K., Bailey D.H., Thompson C.A., Siegler R.S. (2014). Relations of different types of numerical magnitude representations to each other and to mathematics achievement. J. Exp. Child Psychol..

[48] Chen Q., Li J. (2014). Association between individual differences in non-symbolic number acuity and math performance: A meta-analysis. Acta Psychol..

[49] Chu F.W., van Marle K., Geary D.C. (2015). Early numerical foundations of young children’s mathematical development. J. Exp. Child Psychol..

[50] Hyde D.C., Khanum S., Spelke E.S. (2014). Brief non-symbolic approximate number practice enhances subsequent exact symbolic arithmetic in children. Cognition.

[51] Obersteiner A., Reiss K., Ufer S. (2013). How training on exact or approximate mental representations of number can enhance first grade students basic number processing and arithmetic skills. Learn. Instr..

[52] Park J., Brannon E.M. (2013). Training the approximate number system improves math proficiency. Psychol. Sci..

[53] Mussolin C., Nys J., Content A., Leybaert J. (2014). Symbolic number abilities predict later approximate number acuity in preschool children. PLoS ONE.

[54] Opfer J., Siegler R.S., Holyoak K.J., Morrison R.G. (2012). Development of quantitative thinking. The Oxford Handbook of Thinking and Reasoning.

[55] Piazza M., Pica P., Izard V., Spelke E., Dehaene S. (2013). Education enhances the acuity of the nonverbal approximate number system. Psychol. Sci..

[56] Carey S., Shusterman A., Haward P., Distefano R. (2017). Do analog number representations underlie the meanings of young children verbal numerals?. Cognition.

[57] Wagner J.B., Johnson S.C. (2011). An association between understanding cardinality and analog magnitude representations in preschoolers. Cognition.

[58] Odic D., Le Corre M., Halberda J. (2015). Children’s mappings between number words and the approximate number system. Cognition.

[59] Gunderson E.A., Spaepen E., Levine S.C. (2015). Approximate number word knowledge before the cardinal principle. J. Exp. Child Psychol..

[60] Ansari D., Donlan C., Thomas M.S.C., Ewing S.A., Peen T., Karmiloff-Smith A. (2003). What makes counting count? Verbal and visuo-spatial contributions to typical and atypical number development. J. Exp. Child Dev..

[61] Van Herwegen J., Ansari D., Xu F., Karmiloff-Smith A. (2008). Small and large number processing in infants and toddlers with Williams syndrome. Dev. Sci..

[62] Dehaene S., Bossini S., Giraux P. (1993). The mental representation of parity and number magnitude. J. Exp. Psychol. Gen..

[63] Spaepen E., Coppola M., Spelke E.S., Carey S.E., Goldin-Meadow S. (2011). Number without a language model. Proc. Natl. Acad. Sci. USA.

[64] Siegler R.S., Jenkins E. (1989). How Children Discover New Strategies.

[65] Mandler J. (1992). How to build a baby: II. Conceptual primitives. Psychol. Rev..

[66] Gelman S., Meyer M. (2011). Child categorization. WIREs Cogn. Sci..

[67] Molenaar P.C.M. (2004). A manifesto on psychology as idiographic science: Bringing the person back into scientific psychology—This time forever. Measurement.

[68] Dowker A. (2008). Individual differences in numerical abilities in preschoolers. Dev. Sci..

[69] Kolkman M.E., Kroesbergen E.H., Leseman P.P.M. (2013). Early numerical development and the role of non-symbolic and symbolic skills. Learn. Instr..

[70] Nesselroade J.R., Gerstorf D., Hardy S.A., Ram N. (2007). Idiographic filters for psychological constructs. Measurement.

